# Systemic Inflammation Predicts All-Cause Mortality: A Glasgow Inflammation Outcome Study

**DOI:** 10.1371/journal.pone.0116206

**Published:** 2015-03-02

**Authors:** Michael J. Proctor, Donald C. McMillan, Paul G. Horgan, Colin D. Fletcher, Dinesh Talwar, David S. Morrison

**Affiliations:** 1 Academic unit of Surgery, School of Medicine-University of Glasgow, Glasgow, United Kingdom; 2 Department of Clinical Biochemistry, Royal Infirmary, Glasgow, United Kingdom; 3 West of Scotland Cancer Surveillance Unit, University of Glasgow, Glasgow, United Kingdom; Universtiy of Maryland Schoool of Medicine, UNITED STATES

## Abstract

**Introduction:**

Markers of the systemic inflammatory response, including C-reactive protein and albumin (combined to form the modified Glasgow Prognostic Score), as well as neutrophil, lymphocyte and platelet counts have been shown to be prognostic of survival in patients with cancer. The aim of the present study was to examine the prognostic relationship between these markers of the systemic inflammatory response and all-cause, cancer, cardiovascular and cerebrovascular mortality in a large incidentally sampled cohort.

**Methods:**

Patients (n = 160 481) who had an incidental blood sample taken between 2000 and 2008 were studied for the prognostic value of C-reactive protein (>10mg/l, albumin (>35mg/l), neutrophil (>7.5×10^9^/l) lymphocyte and platelet counts. Also, patients (n = 52 091) sampled following the introduction of high sensitivity C-reactive protein (>3mg/l) measurements were studied. A combination of these markers, to make cumulative inflammation-based scores, were investigated.

**Results:**

In all patients (n = 160 481) C-reactive protein (>10mg/l) (HR 2.71, p<0.001), albumin (>35mg/l) (HR 3.68, p<0.001) and neutrophil counts (HR 2.18, p<0.001) were independently predictive of all-cause mortality. These associations were also observed in cancer, cardiovascular and cerebrovascular mortality before and after the introduction of high sensitivity C-reactive protein measurements (>3mg/l) (n = 52 091). A combination of high sensitivity C-reactive protein (>3mg/l), albumin and neutrophil count predicted all-cause (HR 7.37, p<0.001, AUC 0.723), cancer (HR 9.32, p<0.001, AUC 0.731), cardiovascular (HR 4.03, p<0.001, AUC 0.650) and cerebrovascular (HR 3.10, p<0.001, AUC 0.623) mortality.

**Conclusion:**

The results of the present study showed that an inflammation-based prognostic score, combining high sensitivity C-reactive protein, albumin and neutrophil count is prognostic of all-cause mortality.

## Introduction

There is now good evidence that markers of the systemic inflammatory response, namely C-reactive protein and albumin using standard thresholds (termed the Glasgow Prognostic Score, GPS) have independent prognostic value in patients with a variety of cancers [1]. Similarly, neutrophils and lymphocytes using standard thresholds (termed the neutrophil lymphocyte ratio, NLR) have also been shown to have independent prognostic value [2]. Recently, in an effort to rationalise and consolidate the literature we have examined the relationship between these markers of the systemic inflammatory response, together with high sensitivity C-reactive protein measurements and platelet counts, and survival in more than 12,000 cancer patients. This resulted in an optimised score (termed the optimised Glasgow Prognostic Score, oGPS) composed of high sensitivity C-reactive protein (>3mg/l), albumin (<35g/l), neutrophil (>7.5 x 10^9^) and platelet (>400 x 10^9^) counts that had a superior predictive value when compared with the established GPS [3]. It remains to be determined whether such markers and scores also are associated with survival in other disease states.

It is therefore of interest that there have been numerous reports investigating the relationship between an elevated C-reactive protein concentration and increased risk of cardiovascular [4–6], cerebrovascular [5,7] and all-cause mortality [8,9]. There have also been occasional reports of other markers of the systemic inflammatory response, including albumin [10,11], neutrophil count [12], lymphocyte count [13,14] and platelet count [15] being associated with all-cause mortality.

Therefore, the aim of the present study was to examine the relationship between markers of the systemic inflammatory response and all-cause, cancer, cardiovascular and cerebrovascular mortality, in a large incidental cohort.

## Materials and Methods

### Study design

From the Glasgow Inflammation Outcome Study (GIOS) cohort previously described, patients in North Glasgow, who had a single blood sample taken for C-reactive protein and albumin were considered [16]. Only those patients who also had a differential white cell count available, including lymphocyte, neutrophil and platelet count were included. Patients were sampled incidentally between January 2000 and October 2008. The sample size was originally based on similar work investigating the association between C-reactive protein and all-cause survival [8]. If multiple samples had been taken during this time period, only the initial set was used. Only those patients undergoing blood sampling after the introduction of the routine measurement of high sensitivity C-reactive protein (1^st^ of August 2006), were included in the analysis investigating the prognostic value of high sensitivity thresholds (≤5mg/l). As the presence of malignancy is known to be associated with activation of the systemic inflammatory response [16], all patients with a diagnosed malignancy any time prior to or within 14 days of blood sample were noted and this factor was included in the analysis. Whether the sample was taken as an outpatient or inpatient was also noted. All malignancies were included except non-malignant melanoma skin cancers.

Patient outcome and mortality was established through linkage with the Information Services Division for Scotland (ISD). Deaths were classed as all-cause and further subdivided into cancer specific, cardiovascular and cerebrovascular. Patients were excluded if under the age of 16 years, demographic details were incomplete or if returned ISD fields were inaccurate or incomplete. Ethical approval was granted for the present study by the Research Ethics Committee, North Glasgow NHS Trust.

### Methods

Patients with routine laboratory measurements of C-reactive protein, albumin and a differential white cell count, including neutrophil, lymphocyte and platelet counts were obtained by systematically searching the North Glasgow biochemical and haematological database systems. As previously reported, 223 303 patients were identified [16]. The limit of detection of C-reactive protein was a concentration of less than 5mg/l up to 1^st^ of August 2006 and 0.05mg/l thereafter. Standardised thresholds for C-reactive protein (>10mg/l), neutrophil count (>7.5×10^9^/l), lymphocyte count (>3×10^9^/l) and platelet count (>400×10^9^/l) were studied. High sensitivity C-reactive protein threshold >3mg/l, previously reported to be associated with survival [17–20] and shown to be the optimal threshold in this cohort [3], was also studied. From previously published work on the present cohort, cumulative scores based on markers of the systemic inflammatory response, were reported to predict cancer outcomes [21]. These scores were simply composed of variables with similar hazard ratios that had previously been validated in cancer cohorts, including the mGPS comprising of C-reactive protein and albumin, as well as the addition of other haematological markers of the systemic inflammatory response including the constituents of a differential white cell count, in particular the neutrophil count, and a high sensitivity C-reactive protein measurement. In the present analysis a model was built up in a similar way for all-cause mortality [3]. Only components that were independently predictive of outcome were taken forward to be included in the final score. The benefit of using high sensitivity C-reactive protein measurements was also investigated by substituting the standard threshold (>10mg/L) with >3mg/L.

Patient outcome and mortality was established through linkage with the ISD for Scotland by exact matching surname, forename, sex and date of birth. If no match on surname then Daitch-Mokotoff followed by NYSIIS soundex algorithms were employed. On remaining unmatched cases, forenames and surnames were switched followed by matching on previous surnames, reversing gender, using first initial opposed to full forename and finally on surname gender and date of birth only. On all those not with exact matches a manual check on whether to include or exclude was taken. Of the 223 303 patients originally identified, 213 127 were successfully matched through the ISD linkage and 10 176 were not identified (5%). Of these matched patients, 160 481 patients had C-reactive protein and albumin as well as a complete differential white cell count. The presence of SMR01 records were used to determine whether a patient was sampled during a hospital admission as well as whether the patient had died. Hospital admission was defined as having been sampled during a continuous inpatient stay. SMRO6 records containing International Classification of Disease (ICD) codes were used to identify those with a diagnosis of cancer prior to, or within 14 days of blood sampling, as previously described [16]. Cancer related mortality was defined as ICD10 codes C00—C97, cardiovascular (ischaemic heart disease) related mortality was defined as ICD10 codes I20—I25 and cerebrovascular disease related mortality was defined as ICD10 codes I60—I69 and established through ISD. At the time of data collection, the ISD held complete SMR01 data on registered deaths until May 2011. Survival was calculated from time of blood sample to date of death or censor date (31st of May 2011).

The Scottish Index of Multiple Deprivation (SIMD) 2006, as recommended by the ISD on behalf of NHS Scotland and the Scottish Government Department of Health, was used to measure deprivation [22]. The SIMD is a validated area-based measure of relative socio-economic circumstance that uses seven domains (current income; employment; health; education, skills and training; geographic access to services; housing; and crime) to score small areas (data zones). Scotland is divided into 6,505 data zones and these have been ranked from 1 (most deprived) to 6,505 (least deprived). These have further been divided into quintiles of the Scottish population; we used quintiles that range from 1 (least deprived) to 5 (most deprived).

### Statistics

The relationships between patient demographics and outcome were analysed using the Pearson’s chi-square test. Cox proportional hazard model was used to analyse the relationship between patient demographics, hospital admission, the presence of cancer, C-reactive protein, albumin, neutrophil, lymphocyte and platelet counts. In those patients with a high sensitivity C-reactive protein measurement, the prognostic value of the combination of C-reactive protein, albumin and neutrophils, adjusted for age, sex, deprivation, hospital admission and the presence of cancer, was analysed using the Cox proportional hazard model and the Area Under the receiver operating characteristic Curve (AUC). During this analysis the proportionality assumptions in the Cox model were explored using a log minus log visual inspection and were found to be satisfactory. The relationship between the optimised Glasgow Prognostic Score and survival was assessed using the Kaplan-Meier log rank. A p value of <0.05 was considered to have statistical significance in the analysis. Analysis was performed using SPSS software (SPSS, Chicago, IL, USA).

## Results

From GIOS cohort of 223 303 patients originally described [16], 209 148 (94%) were matched with a unique patient record in the ISD dataset. Of this group 160 481 fitted the inclusion criteria and had a differential white cell count available, including neutrophil, lymphocyte and platelet counts. Most patients were under 65 years of age (n = 103 779, 65%) Increasing age resulted in increasing mortality with malignancy being the predominant cause of death in all age groups (p<0.001). Most patients were female (n = 85 308, 53%) with significantly increased mortality from cerebrovascular disease (p<0.001) but decreased mortality from malignancy and ischaemic heart disease (both p<0.001) when compared to males. Forty four percent of patients (n = 71 156) were SIMD 5 (most deprived) with mortality levels generally increasing from malignancy, cerebrovascular disease and ischaemic heart disease observed with increasing deprivation. On follow up of all patients, there were 42 242 deaths in total of which 13 176 (31%) were cancer related, 6076 (14%) were cardiovascular related and 3638 (9%) were cerebrovascular related. The minimum follow up from blood sample for all patients was 31 months and the maximum 134 months (median follow up 69 months for survivors). The relationship between patient demographics, hospital admission, history of cancer, C-reactive protein (>10mg/l), albumin, neutrophil, lymphocyte and platelet counts and survival (n = 160 481) is shown in Table 1. Increasing age, male gender, increasing deprivation, hospital admission, history of cancer, high C-reactive protein (>10mg/l), neutrophil and platelet counts were independently associated with an increase in all-cause mortality (all p<0.001). Low albumin and lymphocyte counts were independently associated with an increase is all-cause mortality (all p<0.001). Increasing age, male gender, history of cancer, high C-reactive protein (>10mg/l), neutrophil and platelet counts were independently associated with an increase in cancer specific mortality (all p<0.001). Low albumin and lymphocytes were independently associated with an increase in cancer specific mortality (all p<0.001). Increasing age, male gender, increasing deprivation, hospital admission, history of cancer, high C-reactive protein (>10mg/l), neutrophil and platelet counts were independently associated with an increase in cardiovascular mortality (all p = 0.001). Low albumin was independently associated with an increase in cardiovascular mortality (all p<0.001). Increasing age, hospital admission, no history of cancer, high C-reactive protein (>10mg/l), neutrophil count were independently associated with an increase in cerebrovascular mortality (all p<0.001). Low albumin was independently associated with an increase in cerebrovascular mortality (all p<0.001).

**Table 1:** The relationship between patient demographics, markers of the systemic inflammatory response and mortality in an incidental cohort (n = 160 481).

			All-cause mortality				Cancer mortality				Cardiovascular mortality				Cerebrovascular mortality			
			Deaths = 42 242				Deaths = 13 176				Deaths = 6076				Deaths = 3638			
			Univariate		Multivariate		Univariate		Multivariate		Univariate		Multivariate		Univariate		Multivariate	
		n (%)	HR	p-value	HR	p-value	HR	p-value	HR	p-value	HR	p-value	HR	p-value	HR	p-value	HR	p-value
Age	<65/	103 779 (65)	1		1		1		1		1		1		1		1	
	65–74	26 989(17)	4.00	<0.001	3.31	<0.001	3.76	<0.001	2.21	<0.001	5.80	<0.001	5.64	<0.001	8.45	<0.001	8.18	<0.001
	>75	29 716 (18)	8.27	<0.001	6.51	<0.001	4.36	<0.001	2.41	<0.001	12.58	<0.001	12.36	<0.001	28.41	<0.001	26.10	<0.001
Sex	Male	75 173 (47)	1		1		1		1	<0.001	1	<0.001	1	<0.001	1	<0.001	1	0.232
	Female	85 308 (53)	0.86	<0.001	0.77	<0.001	0.76	<0.001	0.70		0.72		0.63		1.18		0.94	
SIMD	1	21 362 (13)	1		1		1				1		1		1			
	2	17 778 (11)	1.03	0.216	1.11	<0.001	1.03	0.459			1.10	0.104	1.21	0.001	0.84	0.012		
	3	20 432 (13)	1.16	<0.001	1.20	<0.001	1.18	<0.001			1.18	0.002	1.28	<0.001	0.91	0.139		
	4	29 753 (19)	1.28	<0.001	1.30	<0.001	1.23	<0.001			1.42	<0.001	1.47	<0.001	1.00	0.986		
	5	71 156 (44)	1.25	<0.001	1.36	<0.001	1.00	0.882			1.32	<0.001	1.45	<0.001	1.16	0.004		
Hospital Admission	No	74 142 (46)	1		1		1		1		1		1		1		1	
	Yes	86 339 (54)	1.75	<0.001	1.21	<0.001	1.55	<0.001	1.00	0.861	1.81	<0.001	1.33	<0.001	2.00	<0.001	1.49	<0.001
History of Cancer	None	139 080 (87)	1		1		1		1		1		1		1		1	
	Present	21 401 (13)	3.40	<0.001	2.13	<0.001	12.01	<0.001	8.94	<0.001	1.45	<0.001	0.84	<0.001	1.58	<0.001	0.84	<0.001
C-reactive protein	<10mg/l	94 544 (59)	1		1	<0.001	1	<0.001	1	<0.001	1	<0.001	1	<0.001	1	<0.001	1	<0.001
	>10mg/l	65 937 (41)	2.71	<0.001	1.60		3.16		1.85		2.33		1.54		2.17		1.31	
Albumin	>35mg/l	135 087 (84)	1		1	<0.001	1	<0.001	1	<0.001	1	<0.001	1	<0.001	1	<0.001	1	<0.001
	<35mg/l	25 394 (16)	3.68	<0.001	2.02		4.25		2.08		2.36		1.33		2.54		1.41	
Neutrophil count	<7.5×10^9^/l	127 736 (80)	1		1	<0.001	1	<0.001	1	<0.001	1	<0.001	1	<0.001	1	<0.001	1	<0.001
	>7.5×10^9^/l	32 745 (20)	2.18	<0.001	1.49		2.51		1.41		1.89		1.51		1.89		1.52	
Lymphocyte count	>3×10^9^/l	12 542 (8)	1		1	<0.001	1	<0.001	1	<0.001	1	<0.001	1	0.564	1	<0.001	1	0.272
	<3×10^9^/l	147 939 (92)	1.87	<0.001	1.21		1.84		1.19		1.58		0.97		2.00		1.11	
Platelet count	<400×10^9^/l	143 431 (89)	1		1	<0.001	1	<0.001	1	<0.001	1	<0.001	1	<0.001	1	<0.001	1	0.584
	>400×10^9^/l	17 050 (11)	1.64	<0.001	1.07		2.03		1.39		1.17		0.81		1.51		1.03	

The relationship between patient demographics, hospital admission, history of cancer, C-reactive protein (>3mg/l), albumin, neutrophil, lymphocyte and platelet counts and survival in patients with a differential white cell count following the introduction of high sensitivity C-reactive protein (n = 52 091) is shown in Table 2. Increasing age, male gender, hospital admission, history of cancer, high C-reactive protein (>3mg/l), neutrophil and platelet counts were independently associated with an increase in all-cause mortality (all p<0.001). Low albumin and lymphocyte counts were independently associated with an increase in all-cause mortality (all p<0.001). Increasing age, male gender, history of cancer, high C-reactive protein (>3mg/l), neutrophil and platelet counts were independently associated with an increase in cancer mortality (all p<0.001). Low albumin and lymphocyte counts were independently associated with an increase in cancer mortality (all p<0.001). Increasing age, male gender, hospital admission, history of cancer, high C-reactive protein (>3mg/l) and neutrophil counts were independently associated with an increase in cardiovascular mortality (all p<0.001). Low albumin was independently associated with an increase in cardiovascular mortality (p<0.001). Increasing age, hospital admission, history of cancer, high C-reactive protein (>3mg/l) and neutrophil counts were independently associated with an increase in cerebrovascular mortality (all p<0.05). Low albumin was independently associated with an increase in cerebrovascular mortality (p<0.05).

**Table 2:** The relationship between patient demographics, markers of the systemic inflammatory response (including high sensitivity C-reactive protein) and mortality in an incidental cohort (n = 52 091).

			All-cause mortality				Cancer mortality				Cardiovascular mortality				Cerebrovascular mortality			
			Deaths = 8243				Deaths = 3480				Deaths = 929				Deaths = 553			
			Univariate		Multivariate		Univariate		Multivariate		Univariate		Multivariate		Univariate		Multivariate	
		n (%)	HR	p-value	HR	p-value	HR	p-value	HR	p-value	HR	p-value	HR	p-value	HR	p-value	HR	p-value
Age	<65/	36 834 (71)	1		1		1		1		1		1		1		1	
	65–74	7277 (14)	4.28	<0.001	2.95	<0.001	4.36	<0.001	2.12	<0.001	6.26	<0.001	5.73	<0.001	7.75	<0.001	7.39	<0.001
	>75	7880 (15)	8.10	<0.001	5.24	<0.001	4.82	<0.001	2.20	<0.001	14.36	<0.001	13.18	<0.001	24.31	<0.001	22.20	<0.001
Sex	Male	25 411 (49)	1	<0.001	1	<0.001	1	<0.001	1	<0.001	1	<0.001	1	<0.001	1	0.058		
	Female	26 680 (51)	0.87		0.77		0.86		0.76		0.72		0.61		1.18			
SIMD	1	6943 (13)	1				1				1				1			
	2	5972 (12)	1.07	0.141			1.02	0.768			1.36	0.024			0.92	0.639		
	3	6749 (13)	1.16	0.001			1.19	0.004			1.23	0.133			0.81	0.199		
	4	9583 (18)	1.17	<0.001			1.07	0.275			1.58	<0.001			0.87	0.355		
	5	22 844 (44)	1.06	0.129			0.83	<0.001			1.21	0.092			0.96	0.777		
Hospital Admission	No	23 616 (45)	1		1		1		1		1		1		1		1	
	Yes	28 475 (55)	1.76	<0.001	1.16	<0.001	1.56	<0.001	0.96	0.229	1.82	<0.001	1.29	<0.001	2.31	<0.001	1.74	<0.001
History of Cancer	None	20 917 (40)	1		1		1		1		1		1		1		1	
	Present	31 174 (60)	4.74	<0.001	2.49	<0.001	12.75	<0.001	8.00	<0.001	1.57	<0.001	0.74	0.002	1.78	<0.001	0.77	0.032
C-reactive protein	≤3mg/l	20 917 (40)	1	<0.001	1	<0.001	1	<0.001	1	<0.001	1	<0.001	1	<0.001	1	<0.001	1	0.032
	>3mg/l	31 174 (60)	3.05		1.55		3.88		1.81		2.78		1.66		2.10		1.25	
Albumin	≥35mg/l	41 010 (79)	1	<0.001	1	<0.001	1	<0.001	1	<0.001	1	<0.001	1	<0.001	1	<0.001	1	0.010
	<35mg/l	11 081 (21)	5.19		2.68		6.40		2.95		3.57		1.87		2.69		1.28	
Neutrophil count	≤7.5×10^9^/l	41 617 (80)	1	<0.001	1	<0.001	1	<0.001	1	<0.001	1	<0.001	1	<0.001	1	<0.001	1	<0.001
	>7.5×10^9^/l	10 474 (20)	2.41		1.63		2.66		1.47		1.80		1.49		2.14		1.89	
Lymphocyte count	>3×10^9^/l	5391 (10)	1	<0.001	1	<0.001	1	<0.001	1	<0.001	1	0.008	1	0.083	1	0.001	1	0.992
	≤3×10^9^/l	46 700 (90)	2.06		1.25		2.42		1.42		1.37		0.80		1.78		1.02	
Platelet count	≤400×10^9^/l	47 160 (91)	1	<0.001	1	<0.001	1	<0.001	1	<0.001	1	0.001	1	0.055	1	<0.001	1	0.950
	>400×10^9^/l	4931 (9)	2.05		1.16		2.70		1.54		1.39		0.82		1.66		1.01	

**Fig 1:**
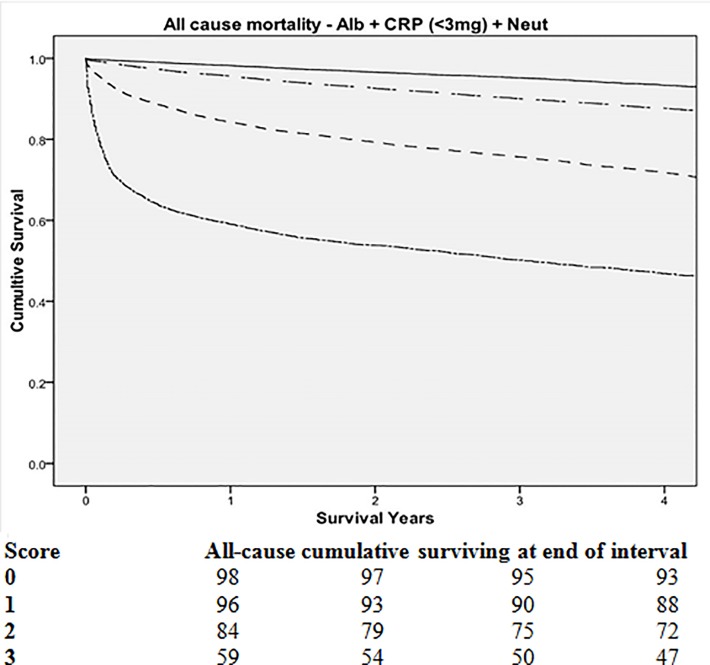
The relationship between cumulative markers of the systemic inflammatory response (including high sensitivity C-reactive protein, albumin and neutrophil count) and survival in an incidental cohort (0-top, solid line; 1-upper separated dot and dash line, 2-middle large dash line, 3-lower close dot and dash line, n = 52 091).

As C-reactive protein, albumin and neutrophils were consistently predictive of all-cause, cancer, cardiovascular and cerebrovascular specific mortality, these were taken forward to examine the prognostic value of their combination. These items were not individually weighted as they had similar predictive value in multivariate analysis and the focus of this process was to provide a simple score easily adopted in the clinical setting. The relationship between cumulative markers of the systemic inflammatory response (including high sensitivity C-reactive protein, albumin and neutrophil count) and mortality (n = 52 091) is shown in Table 3. All combinations of these systemic inflammatory markers were predictive of all-cause, cancer, cardiovascular and cerebrovascular specific survival (all p<0.01) independent of age, sex deprivation, hospital admission and the presence of cancer (Fig. 1). The Kaplan-Meier plot, for a score of 3, shows an initial steep curve that subsequently does not diverge from the curves of a score of 0–2. This suggests that systemic inflammation is particularly predictive of early all-cause mortality. The combination of C-reactive protein (>10mg/L), albumin and neutrophil count had an area under the Receiver Operator Curve (ROC) curve for all-cause mortality of 0.720 (95% CI 0.714–0.727, p<0.001), cancer mortality of 0.728 (95% CI 0.718–0.737, p<0.001), cardiovascular mortality of 0.645 (95% CI 0.627–0.664, p<0.001) and cerebrovascular mortality of 0.612 (95% CI 0.589–0.636, p<0.001). The combination of C-reactive protein (>3mg/L), albumin and neutrophil count had an area under the curve for all-cause mortality of 0.723 (95% CI 0.717–0.729, p<0.001), cancer mortality of 0.731 (95% CI 0.722–0.740, p<0.001), cardiovascular mortality of 0.650 (95% CI 0.633–0.668, p<0.001) and cerebrovascular mortality of 0.623 (95% CI 0.599–0.646, p<0.001). The area under the curve represent the full model for each of the three outcomes.

**Table 3:** The relationship between cumulative markers of the systemic inflammatory response (including high sensitivity C-reactive protein) and mortality in an incidental cohort: Adjusted for age, sex, deprivation, hospital admission and the presence of cancer (n = 52 091).

			All-cause mortality			Cancer mortality			Cardiovascular mortality			Cerebrovascular mortality		
		n (%)	Deaths = 8243			Deaths = 3480			Deaths = 929			Deaths = 553		
			HR	p-value	AUC	HR	p-value	AUC	HR	p-value	AUC	HR	p-value	AUC
	0	26 603 (51)	1		0.720	1		0.728	1		0.645	1		0.612
CRP > 10mg/l	1	13 734 (26)	1.92	<0.001		1.99	<0.001		1.66	<0.001		1.53	<0.001	
+Albumin >35g/L	2	8635 (17)	3.45	<0.001		4.27	<0.001		2.67	<0.001		1.76	<0.001	
+Neutrophils >7.5×10^9^/l	3	3119 (6)	6.45	<0.001		7.95	<0.001		3.49	<0.001		2.50	<0.001	
	0	17 542 (34)	1		0.723	1		0.731	1		0.650	1		0.623
hs-CRP >3mg/l	1	19 880 (38)	1.76	<0.001		1.94	<0.001		1.62	<0.001		1.40	0.006	
+ Albumin >35g/L	2	11 158 (21)	3.58	<0.001		4.44	<0.001		2.92	<0.001		1.90	<0.001	
+ Neutrophils>7.5×10^9^/l	3	3511 (7)	7.37	<0.001		9.32	<0.001		4.03	<0.001		3.10	<0.001	

## Discussion

The results of the present study show that, in a large incidentally sampled cohort of patients (n = 160 481), a number of routinely measured, objective markers of the systemic inflammatory response were independently associated with all-cause mortality. Furthermore, C-reactive protein, albumin and neutrophils were confirmed to have independent prognostic value in patients dying of cancer as well as cardiovascular and cerebrovascular disease. These results implicate the activation of the systemic inflammatory response as a key factor in the shortened survival in common lethal disease states.

The results of the present study are consistent with previous work using individual markers of the systemic inflammatory response to predict all-cause mortality [8–12] and goes further to demonstrate that the combination of C-reactive protein, albumin and neutrophils can further improve the prognostic value of the systemic inflammatory response. Furthermore, these associations remained following adjustment for hospital admission and a previous diagnosis of cancer, events likely to be associated with raised inflammatory markers and reduced survival. It was of interest that a history of cancer, in those dying from cardiovascular and cerebrovascular disease, was positively associated with mortality in univariate survival analysis but negatively associated in multivariate analysis. The basis of this observation is not clear, however, it is worth noting that age was a dominant factor associated with outcome, in particular for cardiovascular and cerebrovascular mortality. Moreover, when all factors were entered into multivariate analysis, the hazard ratios of a number of variables including C-reactive protein and albumin, as well as neutrophil, lymphocyte and platelet counts were reduced. Therefore, it may be that a history of cancer is acting as a surrogate marker for age during univariate analysis with this effect being corrected for in multivariate regression. A similar interaction may explain the observed relationship between platelet count and cardiovascular mortality, as it was noted that as age increased, so did the percentage of patients with a high platelet count (<65 10%, 65–75 12%, >75 13%).

It was also of interest that, for similar incremental increases in individual markers of the systemic inflammatory response, the hazard ratios were greater for cancer mortality, when compared to cardiovascular and cerebrovascular mortality. These results are consistent with those reported by Marsik and co-workers [8] in a hospital based cohort of 274,515 patients using C-reactive protein as a marker of the systemic inflammatory response. The basis of this strong association between the systemic inflammatory response and cancer mortality, when compared with cardiovascular and cerebrovascular mortality, is not clear but highlights the potential of the systemic inflammatory response as a therapeutic oncological target [23,24].

In the present study the variables chosen, and the method of their combination for a cumulative score, was based on previous work establishing the prognostic value of the systemic inflammatory response in patients with cancer [3]. It was noted that while the addition of a high sensitivity C-reactive protein did little to improve the cumulative model's AUC, it did illustrate the value of a C-reactive protein threshold (<3mg/L) commonly used in cardiovascular disease and appeared to better differentiate those patients that were likely to do particularly well. The present Receiver Operator Curve analysis demonstrated relatively wide confidence intervals as well as overlap between the curves for the different combinations of variables and it is possible that these do not represent the optimum combination in cardiovascular and cerebrovascular disease. Nevertheless, the present analysis does produce a functional, easy to use, clinical score that highlights the importance of the systemic inflammatory response in disease states including cancer, cardiovascular and cerebrovascular disease.

The basis of the independent prognostic value of C-reactive protein, albumin and neutrophil count over all disease states (and platelets in cancer) is likely to be complex. However, these factors are known to be involved with regulation of the immune/inflammatory response and in particular the innate immune/inflammatory response. Therefore, it may be hypothesised that the complementary prognostic value of these factors reflects an upregulation of the innate immune/inflammatory responses The consistency of observations across different tumour types and now different disease states suggests that this hypothesis is worthy of further study.

With reference to cancer, the role of markers of the systemic inflammatory response in predicting outcome has become recognised [1,2] and the clinical utility of combining such markers with tumour based factors to provide improved patient counselling and personalised treatment is becoming increasingly recognised [25–27]. The present results provide a framework for the further development of a paradigm that uses both patient and tumour based objective factors to predict patient prognosis.

With reference to cardiovascular and cerebrovascular disease, the prognostic value of C-reactive protein has become increasingly recognised [28]. Indeed, high sensitivity C-reactive protein has been recommended for use in the risk stratification of cardiovascular disease in certain patient groups [29]. Therefore, the present results also provide further evidence for the clinical utility of systemic inflammation-based scoring systems in predicting outcome in cardiovascular and cerebrovascular disease. Indeed, this approach with C-reactive protein and albumin as core factors, has recently been validated in the NHANES III cohort [30] and while the AUCs in the present cohort were around 0.65, this was an improvement on using a single variable alone.

There are striking parallels between the present systemic inflammatory response and the phenomenon of allopathic (also allostatic) load. This ‘wear and tear’ occurs as a consequence of the repeated activation of compensatory physiological mechanisms to chronic stress, including the hyperactivation of the hypothalamic-pituitary axis [31]. As such, the allostatic load can significantly accelerate the ageing process with this being proposed as a mechanism by which deprivation impacts on life expectancy. Therefore, it can be readily envisaged that the systemic inflammatory response may play a key role in the allostatic load paradigm given that measures of the systemic inflammatory response were directly associated with increasing levels of deprivation in the present study. Indeed, it was recently reported that increased physical activity was associated with lower inflammatory scores and allostatic load [32]. These associations are worthy of further study.

The present cohort study has a number of limitations. The patients were selected on the basis that measurements of C-reactive protein, albumin and a differential white cell count had been performed and were therefore not necessarily representative of all patients treated in the North Glasgow area. It is also recognised that the Glasgow based population has reduced life expectancy when compared to the rest of Europe and the present cohort may have had high levels of concurrent morbidity causing derangement of the variables studied. It is also recognised that while the SIMD has been used in an attempt to control for these confounding factors, this may not have been fully effective, and there remains the possibility of bias.

In summary, a number of markers of the systemic inflammatory response, reflecting an optimised Glasgow Prognostic Score, have prognostic utility not only in cancer, but also in cardiovascular and cerebrovascular disease survival. Further work to validate such findings in other large cohorts is warranted.
